# Optimizations of Placenta Extracellular Matrix‐Loaded Silk Fibroin/Alginate 3D‐Printed Scaffolds Structurally and Functionally for Bone Tissue Engineering

**DOI:** 10.1002/elsc.202400085

**Published:** 2025-01-09

**Authors:** Zahra Bashiri, Zahra Khosrowpour, Ali Moghaddaszadeh, Davod Jafari, Sanaz Alizadeh, Hajar Nasiri, Houman Parsaei, Zahra Keshtkaran, Meghdad Abdollahpour‐Alitappeh, Farshad Bargrizaneh, Behzad Rezaei, Sara Simorgh, Mazaher Gholipourmalekabadi

**Affiliations:** ^1^ Endometrium and Endometriosis Research Center Hamadan University of Medical Sciences Hamadan Iran; ^2^ Department of Anatomy, School of Medicine Iran University of Medical Sciences Tehran Iran; ^3^ Omid Fertility & Infertility Clinic Hamedan Iran; ^4^ Department of Pediatrics University of Minnesota Minneapolis Minnesota USA; ^5^ Departement of Biomedical Engineering, Science and Research Branch Islamic Azad University Tehran Iran; ^6^ Oncopathology Research Center Iran University of Medical Sciences Tehran Iran; ^7^ R&D Department Royan Stem Cell Technology Co Tehran Iran; ^8^ Cellular and Molecular Research Center Iran University of Medical Sciences Tehran Iran; ^9^ Nervous System Stem Cells Research Center Semnan University of Medical Sciences Semnan Iran; ^10^ Community Based Psychiatric Care Research Center, Department of Nursing, School of Nursing and Midwifery Shiraz University of Medical Sciences Shiraz Iran; ^11^ Department of Physiology and Pharmacology Pasteur Institute of Iran Tehran Iran; ^12^ Student Research Committee, School of Health Management and Information Sciences Shiraz Universiy of Medical Sciences Shiraz Iran; ^13^ Department of Surgery, School of Medicine Larestan University of Medical Sciences Larestan Iran; ^14^ Department of Tissue Engineering & Regenerative Medicine, Faculty of Advanced Technologies in Medicine Iran University of Medical Sciences Tehran Iran; ^15^ Department of Medical Biotechnology, Faculty of Allied Medicine Iran University of Medical Sciences Tehran Iran

**Keywords:** 3D printed scaffold, alginate/silk fibroin, human placenta

## Abstract

Recent interest has been focused on extracellular matrix (ECM)–based scaffolds totreat critical‐sized bone injuries. In this study, urea was used to decellularize and solubilize human placenta tissue. Then, different concentrations of ECM were composited with 8% alginate (Alg) and 12% silk fibroin (SF) for printing in order to produce a natural 3D construct that resembled bone tissue. The physical and biological features of the printed structures were evaluated entirely in vitro. Finally, a rat model was employed to examine the optimal 3D printed scaffold (5% ECM) as a bone transplant for the healing of cranial bone lesions. The present investigation demonstrated that decellularizing placental tissue fragments led to efficient removal of cell debris. In addition, a remarkable improvement in the printed scaffolds' mechanical and biological properties was observed by increasing the ECM concentration. The histology studies and real‐time PCR results demonstrated the acceleration of bone regeneration in the bone lesions treated with 5%ECM‐SF/Alg at 4 and 8 weeks after implantation. Overall, these results proved that the placental ECM‐printed scaffolds could potentially construct biomimetic grafts to reconstruct significant bone defects and now promise to proceed with clinical studies.

**Abbreviations**
3D: three‐dimensional
Alg: alginate
ALP: alkaline phosphatase
Col1‐a1: collagen type I‐ alpha 1
CSD: critical‐sized defect
DAPI: 4′,6‐diamidino‐2‐phenylindole
dECM: decellularized extracellular matrix
DI: deionized water
EDTA: ethylenediaminetetraacetic acid
H&E: hematoxylin‐eosin
MSCs: mesenchymal stem cells
OCN: osteocalcin
OD: optical density
OPN: osteopontin
PBS: phosphate‐buffered saline
SD: standard deviation
SDS: sodium dodecyl sulfate
SF: silk fibroin
T: Triton X‐100

## 1 Introduction

Bone plays a crucial role in movement, weight‐bearing, and protection of vital organs. Bone lesions caused by accidents are one of the challenges of medical science at present. These lesions may significantly affect the patient's life. In conditions such as extensive fractures and diseases such as bone tumors and bone cysts (resulting in surgery and removal of the damaged area), severe damage is carried out to the bone, and the natural ability of the bone to repair itself is insufficient [1]. Many treatment solutions have been provided to reduce and resolve this problem. Current treatment methods are the use of bone autografts or allografts. Currently, autografts are more widely used, but their access is limited. The place where the bone is removed is usually associated with complications [2].

One of the most promising treatment processes is tissue engineering techniques to reduce bone lesions [3, 4, 5, 6, 7]. Recent research [8, 9] has demonstrated promising outcomes in the creation of biological scaffolds that can offer an environment beneficial to survival, proliferation, and differentiation of resident cells when decellularized extracellular matrix (dECM)–based scaffolds are used for rehabilitation of damaged tissues or organs [10, 11]. Synthetic biomaterials lack cellular recognition signals making dynamic reciprocity between ECM and cells more difficult. Interestingly, ECM is a great material to use for creating scaffolds for tissue engineering because of its flexibility, regardless of where it originates. Placental tissue‐derived ECM is a desirable choice and has several advantages since the ECM continues to regenerate [12]. The human placenta is a rich source of stem cells, growth hormones, and protein that is accessible after delivery [13]. Some products derived from placental grafts have previously been used for tissue regeneration. When they are not decellularized, they're only applied to regions with weak vascularization, such as cartilage, tendons, ligaments, and wounds. Therefore, tissue decellularization is a method for obtaining ECM proteins and preventing an adverse host immune response [14, 15]. The human placenta is completely decellularized by forming an extracellular matrix solution to construct a 3D scaffold. The ability of placental ECM compounds to modulate cellular activity more efficiently than synthetic biomaterials makes them an ideal model for therapeutic applications. This issue has prompted researchers to develop biological platforms for bone regeneration from decellularized placenta [16, 17].

Three‐dimensional (3D) printing is used as a strategy of scaffold construction to develop whole or parts of organs [18]. This technology uses computed tomography to create biological structures containing tissues, cells, and biomolecules to provide suitable treatment for tissue defects. Since 3D printing techniques do not have the problems associated with tissue engineering methods for precise 3D organization, they have been investigated for various biological applications [19, 20, 21]. Wilson et al. used the bioprinting technique for the first time in 2003 to make a scaffold that mainly contained cartilage cells [22]. The biomaterial utilized to create printed platforms is derived from natural or synthetic polymers [23, 24]. To develop dECM‐based bio‐inks, diverse tissues, including fat, brain, cartilage, cornea, heart, kidney, cornea, liver, trachea, testis, and muscle have been decellularized. Preparing dECM‐based bio‐ink involves removing tissue cells while conserving the ECM's native structure and composition. Paying attention to the physical and biochemical characteristics of dECM‐based bio‐inks provides the possibility of imitating the mechanical properties of different tissues. When it comes to 3D printing for tissue engineering, ECM‐based bio‐inks provide the most challenges [25]. Bio‐ink for 3D printing must have appropriate viscosity and mechanical properties to enable structural classification [26, 27]. To overcome the limitations of synthetic scaffolds, different concentrations of placental ECM were composited with silk fibroin (SF) and alginate (Alg) to prepare an optimized printable ECM‐based bio‐ink. SF is a natural polymer with a unique (crystalline) β‐sheet‐rich architecture [28]. Biocompatibility, low inflammatory response, biodegradability, low price, and excellent strength make SF an excellent source for many regenerative processes [25]. Chemical modification of SF can create composites, and its integration with other biological materials can provide a variety of scaffold morphologies. This allows SF scaffolds to be tailored for particular applications [29, 30]. Alg is a natural hydrophilic polysaccharide with guluronic and mannuronic acid units. This material is nontoxic, biodegradable, biocompatible, nonimmunogenic, and usually derived from brown algae and bacteria cell walls [31]. Due to the unique characteristics of this material and the ability to form a hydrogel, alginate is used in the design and manufacture of bio‐inks. It leads to the stabilization of the printed structure [32, 33]. Alg‐SF copolymers can strengthen the internal structure and regulate biodegradation, leading to better performance [28].

In this study, human placental tissue was decellularized and solubilized. The different concentrations of placental ECM were loaded into SF and Alg solution to optimize a 3D bio‐ink. The swelling rate, degradability, mechanical strength (compression and tension), cell adhesion and in vitro biocompatibility assays were carried out to study the 3D printed constructs in vitro and in vivo. Subsequently, an in vivo assay was performed to examine the ability of the optimized 3D printed ECM‐SF/Alg composition to heal critical‐sized rat calvarial bone lesions.

## 2 Materials and Methods

### 2.1 Decellularization and Preparation of Human Placental Tissue

After obtaining consent, placental tissue was taken from mothers who were free of AIDS, hepatitis B and C, syphilis, and other sexually transmitted infections. After that, the tissue was transported to the lab in a sterile saline solution. The placental tissues were fragmented into small segments. Placental tissue was then decellularized using a process described previously [34, 35]. Briefly, the placental fragments have been in contact with Triton X‐100 (T) and sodium dodecyl sulfate (SDS, USA, SDS, Sigma‐Aldrich). This was followed by several washes with distilled water and a week‐long centrifugation. The effectiveness of the placental tissue's decellularization procedure was confirmed by employing staining procedures such as hematoxylin‐eosin (H&E) and 4′,6‐diamidino‐2‐phenylindole (DAPI; Thermo Scientific).

### 2.2 Decellularized Human Placenta Solubilization

Solubilization of the decellularized human placenta was accomplished using a method outlined in our recent study [36]. Briefly, the decellularized placental pieces were treated for 24 h on a magnetic stirrer with a 4 M urea buffer. After that, they spent 20 min centrifuging at ×14,000 rpm. The supernatant was sterilized by adding 2.5 mL of chloroform and 1 L of TBS solution to a dialysis bag. The polymerized proteins were removed by centrifuging the dialysis bag contents at 3000 rpm for 15 min. The viscous supernatant was gathered and kept at −80°C. ECM was vacuum‐dried overnight after frozen at −80°C. The lyophilized sample was kept at −20°C until use.

### 2.3 Manufacture of ECM‐Loaded Silk Fibroin/Alginate 3D‐Printed Structures

#### 2.3.1 Silk Fibroin Extraction

*Bombyx mori* silkworm cocoons were used to extract SF. The cocoons were sliced into fragments and then embedded in a bolning sodium carbonate solution for 1 h. The cocoons were cleaned with deionized water (DI) and then dried at 25°C. The sericin‐free SF fibers were dissolved in a highly concentrated lithium bromide solution for 2 h, then transferred into a dialysis membrane (dialysis bag) and put in a container containing DI while stirring at a steady speed. After that, the SF solution was centrifuged to get rid of the impurities. Finally, the bio‐ink was prepared using the purified SF solution.

#### 2.3.2 ECM Solution Preparation

To determine the optimal ECM concentration for printing scaffolds obtained from placental tissue, ECM powder (1.5%, 3%, and 5%) was added to 1 mL of phosphate‐buffered saline (PBS), followed by magnetic stirring for 24 h to generate the ECM solution. To ensure that there were no residual particles, the desired solution was centrifuged for 10 min at 500 × *g*. The pH value of the ECM solution was regulated to 7 by adding a cold NaOH. After this stage, the solution was kept for 1 week at 4°C.

#### 2.3.3 Bio‐Ink Preparation

A total of 0.12 g of Alg powder was dissolved in 10 mL of PBS to prepare a 12% w/v Alg solution. To create a hybrid bio‐ink, 12% Alg solution and 8% SF solution were blended in a proportion of 1:1. ECM solution in three different concentrations of 0%, 1.5%, and 3%, and 5% w/v were included in the prepared SF/Alg solution to produce ECM bio‐inks. The bio‐inks were centrifuged for 1 min at ×1000 rpm to eliminate any trapped air.

#### 2.3.4 Hydrogel Scaffold 3D Printing

Utilizing CAD/CAM software and a 3DPL Bioprinter (3DPL, Iran), the prepared bio‐inks were printed. The scaffolds were manufactured as a 12 × 12 mm mesh with a string thickness of 2 mm and a spacing of 1 mm. The injector was loaded with 3 mL bioink for each production. The printing conditions were as follows: a 21‐gauge syringe, the lowest pressure (1,1 bar), and speed (2 mm/s). The printing procedure utilized glass microscope slides as the printing surface. To remove excess calcium ions, the scaffolds were washed with deionized water for 30 min, after being crosslinked for 15 min with 300 mM calcium chloride. In the following experimental groups, the structures were 3D printed:

1. **Group 1 (G1)**: SF/Alg and ECM (0wt%) hybrid scaffolds
2. **Group 2 (G2)**: SF/Alg and ECM (1.5wt%) hybrid scaffolds
3. **Group 3 (G3)**: SF/Alg and ECM (3wt%) hybrid scaffolds
4. **Group 4 (G4)**: SF/Alg and ECM (5wt%) hybrid scaffolds

### 2.4 Characterizations of ECM‐SF/Alg 3D‐Printed Structures

#### 2.4.1 Mechanical Behaviors of 3D‐Printed Hydrogel Scaffold

The scaffolds' modulus, compressive, and tensile strengths were determined using a mechanical testing instrument (Hct400/25, Zwick/Roell). The samples of each group were prepared with a 2 mm thickness and a 6 mm height. A universal testing machine was employed to apply a uniaxial compression test with a 10 N load cell and 0.1 mm/s loading speed. The top and bottom surfaces of the specimen were bonded to the compression testing plates to avoid slippage during testing. The specimens were then examined for an 80% strain level. Moreover, the machine elongated the 3D frameworks at a rate of 0.2 mm/s to fail to assess the samples' mechanical strength.

#### 2.4.2 Degradation Rate of 3D‐Printed Hydrogel Structures

All scaffolds were weighed (Md) and then embedded in 3 mL of PBS at 37°C. Every 3 days, the PBS solution was regularly replaced. The samples were collected after 1 h, 1 day, 7 days, 14 days, and 30 days post‐PBS embedding interval times, gently rinsed with DI to eliminate buffer salts, and finally lyophilized. The freeze‐dried scaffolds were weighed (Mf). The percentage of weight loss was calculated using the following formula (Equation 1):

(1) $$\text{Mass}\;\text{loss}\left(\;\;\;\%\;\;\;\right)=\left[\left(\text{Mf-Md}\right)\text{/Md}\right]\;\;\;\times \;\;\;\text{100}$$

#### 2.4.3 Swelling Rate of 3D‐Printed Hydrogel Scaffold

The printed specimens were weighed (Md) and then embedded in 2 mL PBS to estimate the swelling rate of the scaffolds. The scaffolds were subjected to incubation at a temperature of 37°C for 72 h. At each interval of 0.5, 1, 2, 4, 8, 12, 24, 48, and 72 h, the scaffolds were collected and subjected to a filtration process to eliminate any unabsorbed PBS. Measurements of scaffold weight (Mw) were subsequently taken. The percentage swelling rate was calculated as follows (Equation 2):

(2) $$\mathrm{Swelling}\;\mathrm{rate}\left(\%\right)=\left[\left(\mathrm{Mw}-\mathrm{Md}\right)/\mathrm{Md}\right]\times 100$$

### 2.5 In Vitro Cytobiocompatibility

#### 2.5.1 Interaction Between Cell and Scaffold Under SEM

The morphology of mesenchymal stem cells (MSCs) cultured on the 3D printed scaffolds was examined using SEM. In detail, the MSCs (2 × 10^4^ cells/24‐well plates) were seeded onto the hydrogel scaffolds and incubated for 72 h. After that, the media was discarded. The scaffolds were rinsed with PBS. Subsequently, the samples were immersed in 2.5% v/v glutaraldehyde (GA, Sigma‐Aldrich, USA) at 4°C for 4 h. The dehydrating specimens were performed through 50%, 60%, 70%, 80%, 90%, and 100% v/v ethanol concentrations. The construct surfaces were observed under SEM [37].

#### 2.5.2 Cell Metabolic Activity

The viability of the MSCs cultured on the fabricated scaffolds was assessed using an MTT assay. Briefly, after sterilizing the scaffolds using ultraviolet irradiation, samples were incubated for 24 h in the free‐FBS medium to prepare a scaffold extract. 4 × 10^3^ MSCs cells were cultured in a pre‐sterile 96‐well plate contained with 50/50 ratio of extract/DMEM supplemented with 10% FBS and antibiotics. The cells cultured with no scaffold's extract served as control. The cells were incubated to the scaffold extraction for 1, 3, and 7 days. After a predetermined incubation period, the culture media was rinsed with 10% MTT solution and incubated for 4 h. After removing the MTT solution from the wells, dimethyl sulfide was added to dissolve the formazan crystals. Finally, an ELISA plate reader was used to record the optical density (OD) at 570 nm. As a control (100% cell viability), cells were cultivated in a plate with no scaffolds [38]. By the following formula (Equation 3), the percentage of cellular metabolic activity (cell viability) was calculated:

(3) $$\begin{array}{cc} & \mathrm{Cell}\;\mathrm{metabolic}\;\mathrm{activity}\left(\%\right)\\ & =[(\mathrm{Average}\;\mathrm{OD}\;\mathrm{of}\;\mathrm{experiments}-\mathrm{Average}\;\mathrm{OD}\;\mathrm{of}\;\mathrm{control})/\\ & \;\;\;\mathrm{Average}\;\mathrm{OD}\;\mathrm{of}\;\mathrm{control}]\times 100 \end{array}$$

### 2.6 Animal Investigations

#### 2.6.1 Critical‐Sized Bone Defect Model and Implantation

A total of 30 Wistar male rats (250–300 g) were provided from the Pasture Institute (Tehran, Iran). The animals were anesthetized using xylazine and ketamine (100 mg/kg body weight) by intraperitoneal injection. After shaving the head of the rats, the scalpel made a 1.5 cm sagittal incision along the periosteum to create the calvarial bone defect model. A dental drill made an 8 mm nonhealing critical‐sized defect (CSD) in the middle of the skull. Out of the animals, three experimental groups (*n* = 6) were chosen randomly: 0%ECM‐SF/Alg group, 5%ECM‐SF/Alg group, and the control group (bone defect with no treatment). After suture incisions, the animals were fed with antibiotics for 3 days and followed weekly. After 4 and 8 weeks, the rats were sacrificed and the calvariums were harvested for additional examinations.

#### 2.6.2 Histological Evaluation

The implanted bone specimens were collected, fixed in 10% paraformaldehyde, decalcified in 14% ethylenediaminetetraacetic acid (EDTA) for 14 consecutive days, and dehydrated through ethanol solutions. After immersing the specimens in paraffin, serially sectioned with a microtome, and stained with H&E, Masson's trichrome and Alizarin red (Asia Pajhohesh) [39].

#### 2.6.3 Real‐Time PCR

The total RNA was isolated from the bone samples harvested from the implanted sites using RNX‐Plus (SinaClone BioScience, Tehran, Iran), following the manufacturer's guidelines [40]. Three different animals' RNA samples were used to synthesize cDNA using a random hexamer primer; subsequently, RT‐PCR analysis was carried out. Relative quantification expression of collagen type I‐alpha 1 (Col1‐a1), osteopontin (OPN), osteocalcin (OCN), alkaline phosphatase (ALP), and runt‐related transcription factor 2 (RUNX2) was carried out using 2X Q‐PCR Master Mix and the data was calculated using 2^−△△ct^ formula. Primers used are listed in Table 1.

**TABLE 1:** The sequence of primers used in present study for RT‐PCR analysis.

	Gene name	Accession number	Sequence	Product size (bp)
1	ALP	NM_007393.5	F: 5′‐TTAAGGGCCAGCTACACC‐3′ R: 5′‐GATAGGCGATGTCCTTGC‐3′	96
2	Runx2	NM_007742.4	F: 5′‐GAAATGCCTCTGCTGTTA‐3′ R: 5′‐TCTGTCTGTGCCTTCTTG‐3′	165
3	OCN	NM_009930.2	F: 5′‐TCAACAATGGACTTGGAG‐3′ R: 5′‐CAACACATGCCCTAAACG‐3′	177
4	OPN	NM_010612.2	F: 5′‐CCAACTACAACCATGAGAC‐3′ R: 5′‐CATCTGAGTGTTTGCTGTAA‐3′	122
5	COL1	NM_008006.2	F: 5′‐AGAAGAATATGTATCACCAGA‐3′ R: 5′‐AGCAAAGTTTCCAAG‐3′	89
6	HPRT1 (housekeeping gene)	NM_001110268.1	F:5′‐CTTCAGGGATTTGAATCATGTT‐3′ R: 5′‐CGTCGTCTAGTTCTTTACTG‐3′	93

### 2.7 Statistical Analysis

This study used Prism 7 and SPSS for data analysis. Data are expressed as the mean ± the standard deviation (SD). Multiple group studies applied a one‐way ANOVA followed by Tukey's post‐hoc test and a repeated‐measures ANOVA test. In terms of statistics, *p* values less than 0.05 were considered significant.

## 3 Results

### 3.1 Decellularization Efficiency

**FIGURE 1:**
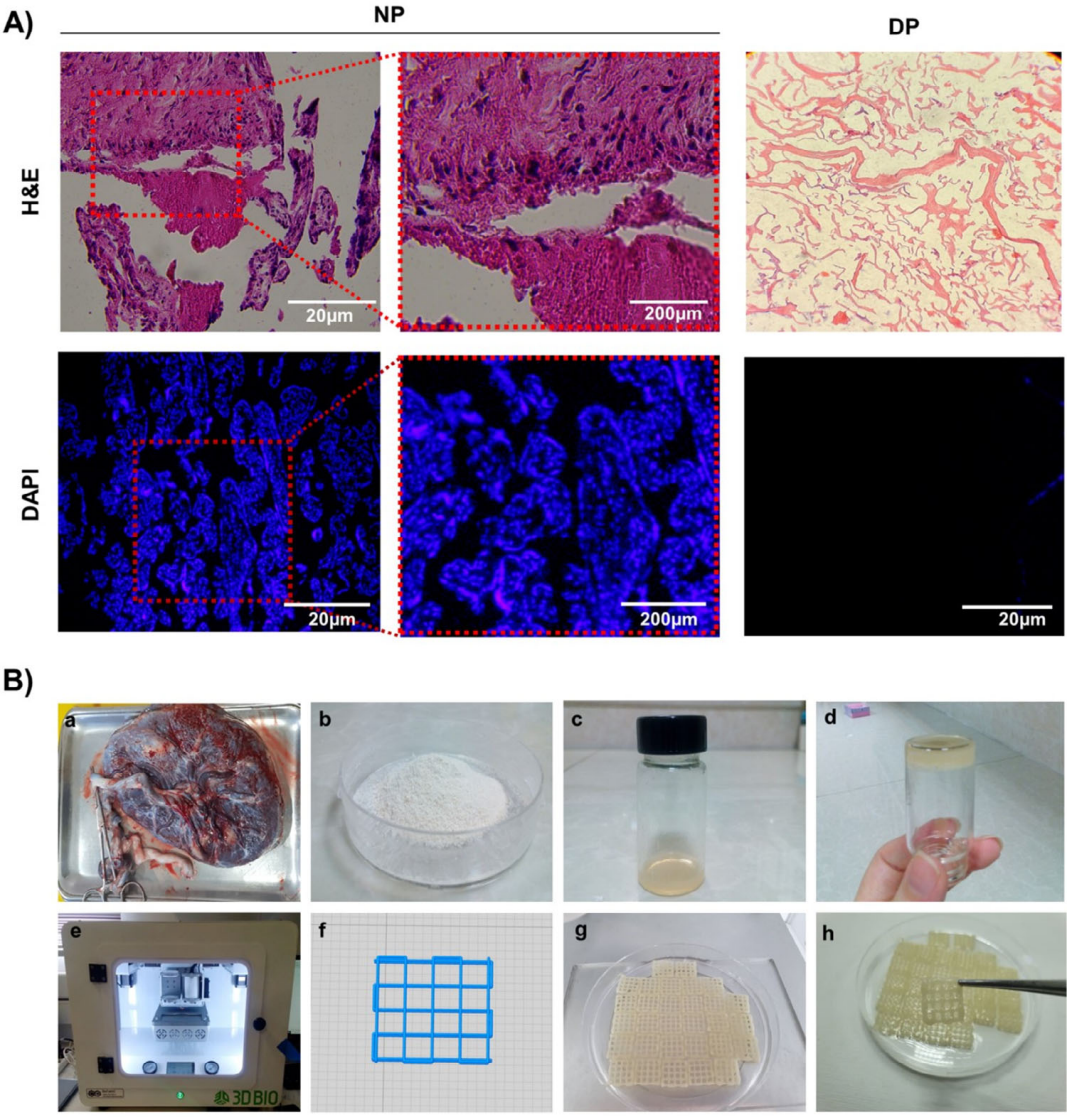
(A) H&E and DAPI assessments of the decellularization procedure in the control group (fresh placenta) and decellularized placental tissue. The cells’ nuclei are indicated with black and white arrows. (B) Steps of preparing the printed hydrogel scaffolds. a: Fresh human placental tissue. b: Extracted ECM powder. c and d: The transformation of an ECM pre‐gel solution derived from human placental tissue from sol (37°C) to gel (25°C). e and f: A scaffold designed in 3D for 3D printing [111]. g: The printed scaffolds before and h: after crosslinking.

H&E and DAPI staining verified the successful cell removal in accordance with Figure 1A. All the findings confirmed the effectiveness of the decellularization protocol in removing cells or genomic DNA. The decellularized and solubilized ECM prepared bio‐ink and fabricated SF/Alg 3D printed scaffolds containing different ECM concentrations. The ECM concentrations >5% w/v were not printable. Figure 1B summarizes scaffold fabrication steps from ECM preparation to 3D printing.

### 3.2 Characterizations of ECM‐SF/Alg 3D‐Printed Structures

#### 3.2.1 Mechanical Behaviors of 3D‐Printed Hydrogel Scaffold

The results of an investigation comparing the compressive and tensile strengths of the printed samples are shown in Tables 2 and 3, respectively. According to the collected data, the average compressive strength of the scaffolds ranged from 1.27 ± 0.31 to 2.24 ± 0.68 MPa. There was a correlation between the concentration of ECM in scaffolds and their compressive modulus. In particular, an increase in ECM concentration increased compression modulus. There were significant differences in stress and Young's modulus between the 5% ECM and the others. Furthermore, a negligible difference in the compressive strength and Young's modulus can be seen between 1.5% and 0%.

**TABLE 2:** Characteristics of the compressive strength of the printed structures.

Group	Ultimate compressive stress (MPa)	Maximum compressive strain (%)	Elastic modulus (MPa) (Young's modulus)
0%ECM‐SF/Alg	1.27 ± 0.31^a^	1.01 ± 0.03^a^	0.721 ± 3.62^a^
1.5%ECM‐ SF/Alg	1.39 ± 0.45^a^	1.20 ± 0.04^a^	0.898 ± 2.59^a^
3%ECM‐ SF/Alg	1.78 ± 0.27^a^	1.66 ± 0.09^a^	1.529 ± 4.43^b^
5%ECM‐ SF/Alg	2.24 ± 0.68^b^	1.88 ± 0.07^a^	1.734 ± 5.18^c^

*Note:* Data from an analysis of compressive strength are reported as mean SD.

^a,b,c^ Values with distinct symbols in each column differ significantly (*p* < 0.05). Each experiment was carried out triple (*n* = 3).

**TABLE 3:** Tensile strength characteristics of the printed structures.

Group	Ultimate tensile stress (MPa)	Maximum tensile strain (%)	Elastic modulus (MPa) (Young's modulus)
0%ECM‐SF/Alg	0.543 ± 0.57^a^	0.651 ± 0.02^a^	0.745 ± 2.63^a^
1.5%ECM‐SF/Alg	0.672 ± 0.42^a^	0.745 ± 0.05^a^	0.873 ± 1.77^a^
3%ECM‐SF/Alg	0.936 ± 0.61^b^	1.27 ± 0.06^b^	1.78 ± 4.26^b^
5%ECM‐SF/Alg	1.289 ± 0.65^b^	1.84 ± 0.08^c^	2.643 ± 4.82^c^

*Note:* Data from an analysis of compressive strength are reported as mean SD.

^a,b,c^ Values with distinct symbols in each column differ significantly (*p* < 0.05). Each experiment was carried out triple (*n* = 3).

In addition, the tensile strength of the printed scaffolds ranged between 1.289 ± 0.56 and 0.543 ± 0.75 MPa. The scaffolds' tensile strength rose dramatically as the ECM content increased. Among the groups examined, those with 0% and 5% ECM exhibited minimum and maximum mechanical strength, respectively. The 5% ECM scaffold group demonstrated superior mechanical properties, which can be attributed to their sophisticated intra‐network architecture and more significant connection, resulting from their smaller pore size. The results showed that 5% of ECM systems had superior mechanical properties than other composite materials.

#### 3.2.2 Degradation Rate of 3D‐Printed Hydrogel Scaffold

**FIGURE 2:**
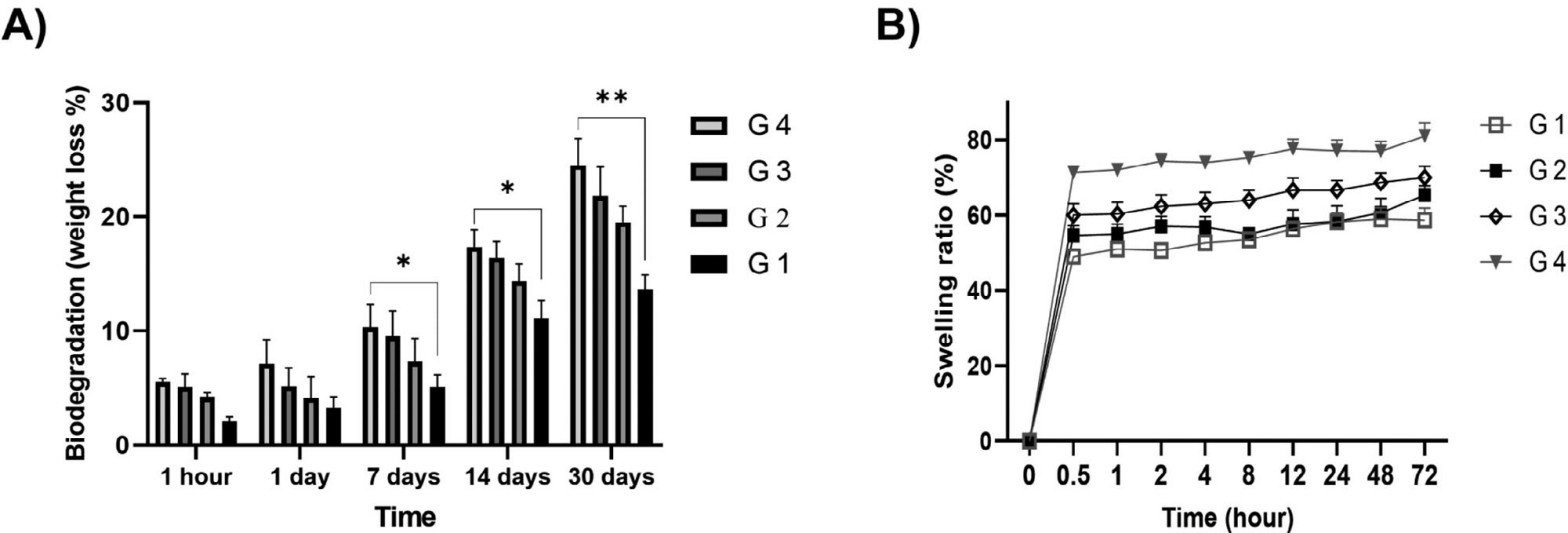
(A) Degradability analysis of the printed scaffold. The data are provided as mean ± SD. There were three repetitions of each test (*n* = 3). (B) Swelling analysis of the printed scaffold. The data are presented as mean ± SD. There were three repetitions of each test (*n* = 3).

After 30 days of incubation, the percentage of degradation rates were 14.06 ± 2.43, 19.53 ± 0.91, 21.84 ± 1.8, and 24.53 ± 2.7 for 0%ECM‐SF/Alg, 1.5%ECM‐SF/Alg, 3%ECM‐SF/Alg, and 5%ECM‐SF/Alg, respectively. The data indicates a correlation between the increase in ECM content and the degradation rate. On days 7, 14, and 30 of incubation, the degradation rate was significantly different between 5%ECM‐SF/Alg (G4) and 0%ECM‐SF/Alg (G1). On the other incubation days, however, there were no obvious distinctions between the experimental groups (*p* >0.05) (Figure 2A).

#### 3.2.3 Swelling Rate of 3D‐Printed Hydrogel Scaffold

The capacity of hydrogel scaffolds to absorb liquids is critical for the transport and penetration of metabolic products, signaling molecules, and nutrients. The water absorption capacity of scaffolds was assessed through a comparative analysis of their pre‐ and post‐immersion weights in distilled water for various time intervals up to 72 h (Figure 2B). The results demonstrated that the composite scaffolds' swelling rate increased when the ECM content increased. The water uptake (%) was 58.66 ± 3.12, 65.33 ± 3.45, 70.04 ± 4.1, and 81.06 ± 4.8 for 0%ECM‐SF/Alg (G1), 1.5%ECM‐SF/Alg (G2), 3%ECM‐SF/Alg (G3), and 5%ECM‐SF/Alg (G4), respectively. This increase was statistically significant between the G4 and G1. This test confirms the degradation data and demonstrates the relationship between the material's greater water absorption capacity and higher hydrolysis sensitivity. As represented in Figure 2B, the hydrogel scaffolds achieved saturation after 30 min; however, the water uptake did not grow substantial until 72 h, and the water absorption did not exceed 85%.

### 3.3 In Vitro Cytobiocompatibility

#### 3.3.1 Interaction Between Cell and Scaffold

**FIGURE 3:**
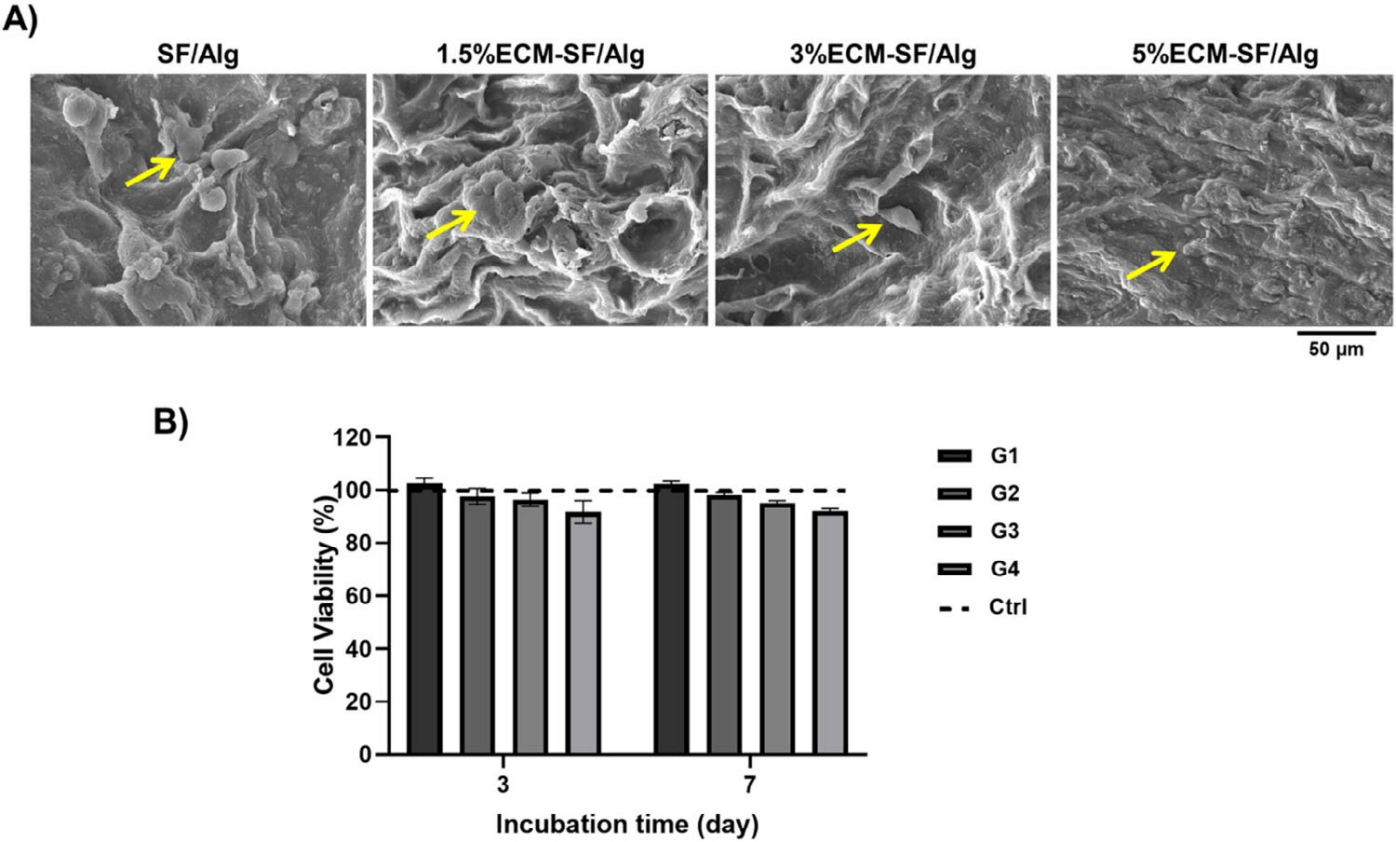
Evaluations of cytobiocompatibility in vitro. (A) The SEM analysis of the MSCs morphology after three days of culture on hydrogels. Yellow arrows indicate MSCs. (B) MTT assay for MSCs seeded onto the scaffolds for 1, 3, or 7 days.

Figure 3A depicts the morphology of MSCs cultured for 3 days on the 3D‐printed constructs. The high attachment and expansion of MSC cells on all the samples were clearly seen.

#### 3.3.2 Cell Metabolic Activity

Figure 3B depicts the metabolic activity of the MSCs grown on the scaffolds. On days 1, 3, and 7 of incubation, the scaffold had no discernible impact on the metabolic activity of the cells in comparison to the control group, which was assumed to have 100% cell vitality.

### 3.4 Animal Study

#### 3.4.1 Histological Evaluation

**FIGURE 4:**
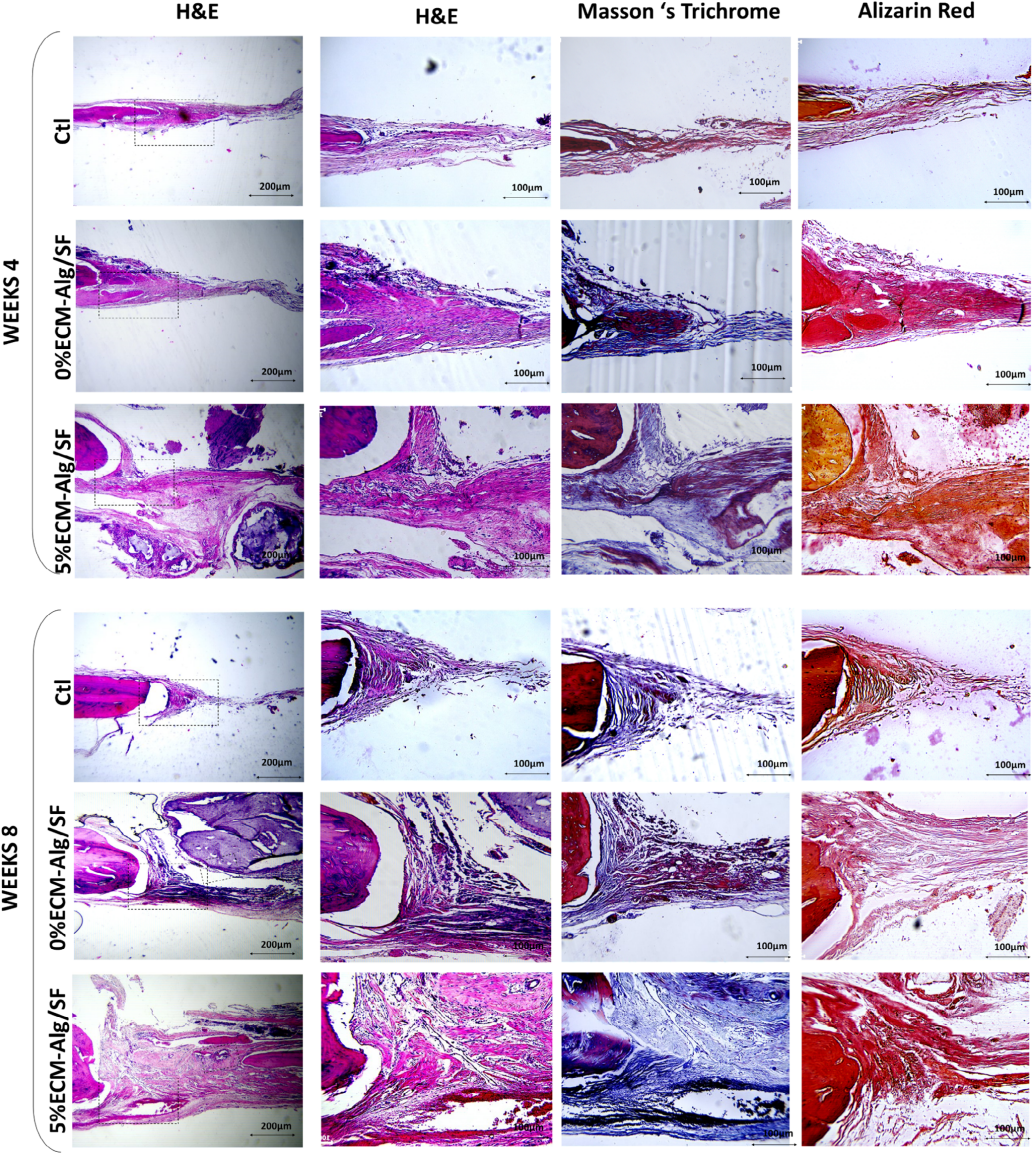
Histological evaluations. H&E, Masson's trichrome, and Alizarin red staining of the bone defect treated with 0%ECM‐SF/Alg, 5%ECM‐SF/Alg and no treatment groups after weeks 4 and 8 post‐implantation. Scale bars 200 and 100 µm. Ctl = control.

Histological findings showed the light connective tissue with fibrin filaments at the defect site in control groups. Still, no remarkable bone production was detected at weeks 4 and 8 (Figure 4). At day 28 post‐treatment, the defect implanted with 5%ECM‐SF/Alg showed significantly thicker connective tissue with a higher rate of new bone formation from the margins, when compared with 0%ECM‐SF/Alg and control groups. At week 8, control groups remained unhealed, whereas both treated defects showed new bone formation. The higher rate of new bone in the bone defects implanted with 5%ECM‐SF/Alg was observed compared to other experimental groups. The dense blue color in MT staining indicates collagen deposition as one of the indicators of new bone regeneration. The amount of collagen was higher in the 5%ECM‐SF/Alg group due to an immature extracellular matrix. According to the Alizarin red staining, calcium deposition in the 5%ECM‐SF/Alg group was more than that in 0%ECM‐SF/Alg group at week 4 and continued to increase at week 8. Histological examinations demonstrated that ECM could accelerate bone formation due to its high collagen and calcium deposition, more than the 0%ECM‐SF/Alg and control group.

#### 3.4.2 Real‐Time PCR

**FIGURE 5:**
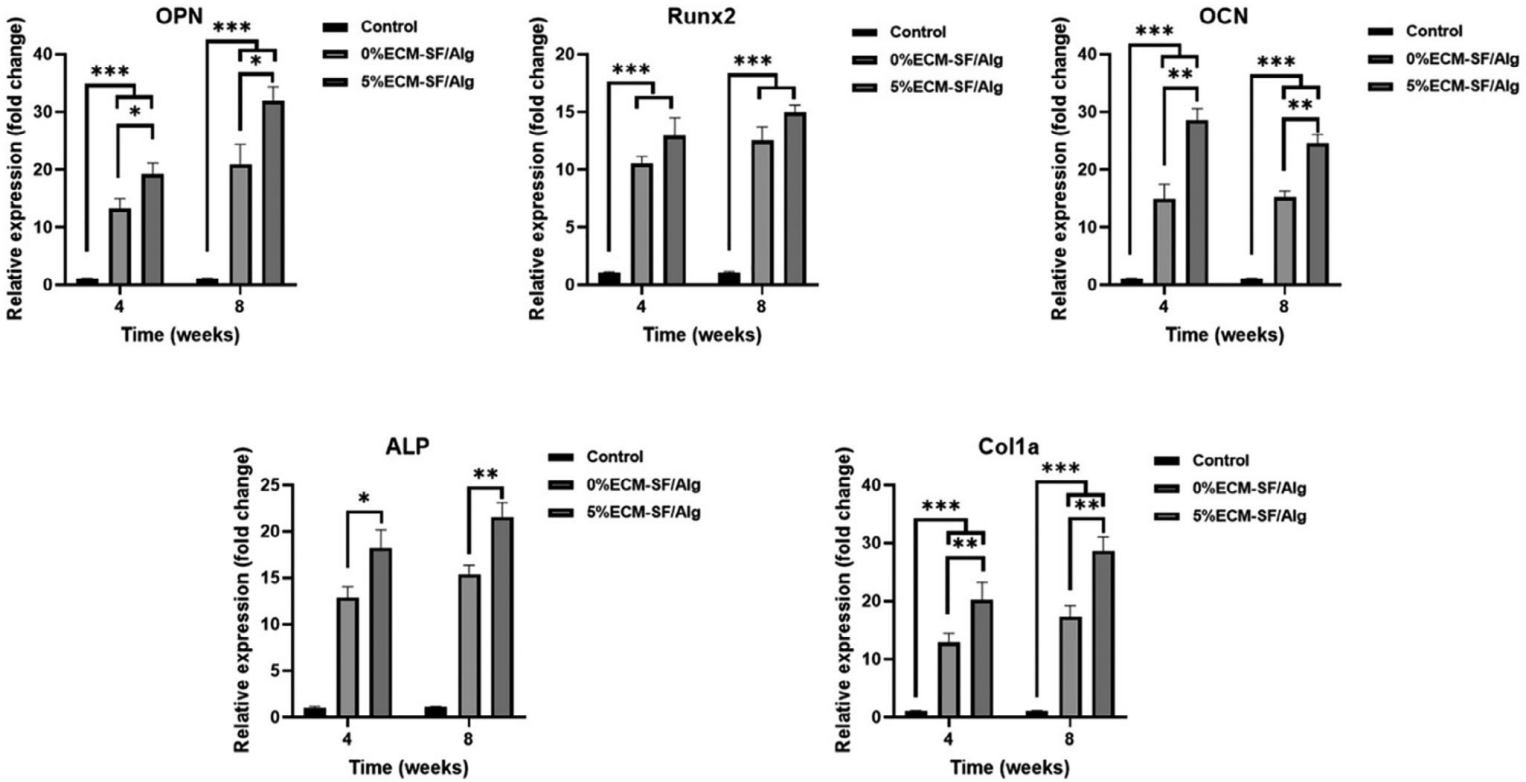
Molecular evaluations of OPN, Runx2, OCN, ALP, and Col1a genes in the bone defects treated with 0%ECM‐SF/Alg, 5%ECM‐SF/Alg, and no treatment. *, **, and *** indicate *p* < 0.05, *p* < 0.001, and *p* < 0001, respectively.

The molecular evaluation of the osteogenesis and bone healing‐related genes in the wounds at weeks 4 and 8 RT‐PCR was performed posttreatment, and the findings are presented in Figure 5. It was seen that both 0%ECM‐SF/Alg and 5%ECM‐SF/Alg induced the expression of osteogenesis bone (ALP, Runx2, OCN, and ALP) and collagen synthesis on weeks 4 and 8 after surgery when compared with the bone defect with no treatment (control group). 5% ECM in the construct markedly affects the expression of bone healing genes at 4 and 8 weeks posttreatment follow‐up.

## 4 Discussion

Nowadays, bone injuries caused by fractures, tumor surgery, or congenital abnormalities are increasing worldwide, and with the age of the world's population, these statistics are alarming [41]. In minor bone defects (with a diameter of less than 6 mm), after a short period, fibroblast cells first proliferate, and then, the graft tissue is formed in the lesion area. Afterward, a bone bridge is created between the two edges of the fracture, and the bone lesion is repaired using the stem cells of the area. However, the formation of a bone bridge is complex in severe bone lesions (with a diameter of more than 6 mm, known as a critical‐sized bone defect), and the repair will not be done well [42]. Bone defects with critical size cannot be treated independently without clinical intervention and can significantly affect the patient's life [4, 43].

The standard treatment for bone regeneration is the use of autograft and allograft. Autograft encounters problems such as limited resources, complications of the donor area, pain, and infection that can affect wound healing. In addition to the abovementioned limitations, allografts also have the risk of immune rejection [44, 45]. Bone tissue engineering has provided an interesting strategy to treating bone deficiency, where patients can receive transplants of natural or artificial bone substitutes [46].

A scaffold with physical and chemical properties similar to bone tissue is used as a bone substitute. Various researchers have shown that a convenient alternative to imitating bone ECM should have biocompatibility, nontoxicity, osteoinductivity, and mechanical features comparable with bone tissue [47, 48]. ECM is a biomaterial originated from natural tissue that has broad preclinical and clinical purposes, including bone and other tissue engineering applications. ECM is composed chiefly of collagen, laminin, fibronectin, proteoglycans, and glycosaminoglycans (GAGs) [49] and profoundly affects cell differentiation [50].

A successful decellularization eliminates cellular and immunogenic agents while conserving the tissue's native ECM components [51, 52]. The decellularization technique was used for the first time in 1973 to treat burn patients [53]. Numerous approaches exist for decellularizing tissues and organs, including chemical, physical, perfusion, and enzymatic [54]. Recently, we have optimized a chemical method for decellularizing human placenta tissue, successfully used for tissue engineering of skin, bone and others [15, 55, 56]. In this method, using SDS/Triton leads to the lysis of cell membranes and the removal of cell debris. Compared with SDS alone, SDS/Triton causes less damage to collagen and GAGs and participates in separating DNA from proteins [57]. Triton is a non‐ionic detergent that is a separating agent in the washing process and does not damage the tissue structure [58, 59]. Evaluation of cellular content showed that the decellularized samples did not have clear nuclei, and the total amount of DNA in the placenta tissue was less than the maximum threshold. Our study confirms other researchers' findings [55, 60].

To form a hydrogel, it is first necessary to convert dECM into soluble protein monomer components. Hydrochloric acid (or acetic acid), pepsin, or urea are used to dissolve ECM. Solubilization using acid leads to the disintegration of collagen intermolecular bonds [61], and the enzyme changes and breaks small to medium molecular weight proteins [62]. According to the study of Moore et al. [63], the human placental tissue ECM extracted by urea maintains proteins, cytokines and growth factors at the physiological level. Also, other studies showed that the urea‐extracted ECM maintained the growth factors necessary for tissue‐specific differentiation [64, 65]. Following prior research [55, 66], the findings of our investigation indicated that urea treatment results in breaking apart more noncovalent bonds and increasing the ECM proteins' solubility. Research indicates that employing water as a solvent speeds up the degradation of scaffolds, as calcium ions are removed from their structure, leading to a loss of mass. In contrast, scaffolds created in PBS buffer experience a slower calcium ion exchange, making the process more challenging [67]. The results of our study also confirmed that the use of PBS solvent causes mechanical stability and reduces the degradability of scaffolds over time, which our results have been confirmed by other studies [68].

Among the methods currently used to fabricate scaffolds, the 3D printing technique significantly impacts the progress of regeneration and bone induction. ECM‐based bi‐oinks for bone tissue engineering have received attention in recent years [69, 70]. ECM‐based bio‐ink is a gel‐like biomaterial with a suitable viscosity for 3D printing to enable injection and hydrogel formation. Some reports have used nanoparticles to increase the viscosity of natural bio‐inks [71, 72]. In this process, nanoparticles such as hydroxyapatite, magnetic iron oxide, gold, and molybdenum‐impregnated bioactive glass can be dispersed in bio‐ink to enhance bone formation. These nanoparticles showed the capability to induce bone and osteoinduction [73].

The stability of bio‐constructs printed using dECM‐based bio‐inks is insufficient due to their poor mechanical properties. In recent years, progress has been made in improving the printing ability of dECM‐based bio‐inks to produce structures with biomechanical properties analogous to those of natural tissues [74]. Other investigations have utilized supporting materials, including PEVA [75], PCL [76], Pluronic F‐127 [77], or crosslinking hydrogels. In another study, vascular tissue‐obtained dECM was combined with alginate to be ionically crosslinked. The CPF127 solution was utilized in the printing of ECM and Alg to facilitate the gelatinization of Alg through the release of Ca^+2^ ions [78].

One of the promising strategies is the combination of dECM components with natural polymers, which have been frequently used in bio‐ink printing [79, 80]. SF is a natural polymer with good mechanical features, low inflammatory reaction, biocompatibility, and high adaptability [81, 82]. SF alone slightly induces osteogenic activity in vivo [83, 84]. Alg is another prominent biopolymer mainly extracted from brown seaweed that regulates SF biodegradation and can improve the internal structure of SF scaffolds [85, 86].

The scaffold's swelling behavior is critical for balancing porosity and mechanical stability during in vivo implantation. Controlled swelling facilitates nutrient and oxygen diffusion, while excessive swelling risks structural integrity [87]. To address this, we optimized the composition of SF, Alg, and placental ECM, stabilizing the bio‐ink's biological structure. By fine‐tuning SF/Alg ratios, we achieved a printable bio‐ink, overcoming challenges such as insufficient viscosity in low concentrations and poor extrudability in high concentrations. Ultimately, 12% Alg and 8% SF combined showed suitable printability to manufacture scaffolds. Many studies have examined the impact of various concentrations of other tissues ECM (0%, 1.5%, 3%, and 5%) on forming 3D‐printed structure [16, 55, 88]. In this study, we used different concentrations of human placenta ECM (0%, 1.5%, 3%, and 5%) to optimize the print. The bioinks contained with more than 5% ECM were not printable. The structural, physical, and biological properties of the 3D‐printed hydrogels were then assessed.

Comparable to natural tissues, hydrogels have elastic properties. The ability of hydrogels to uptake water and degrade in biological systems is one of its benefits when used in tissue engineering. After different time intervals, the swelling behavior of other hydrogels was examined in PBS. The SF/Alg hybrid hydrogel showed a swelling ratio of 58.66 ± 3.12 due to the superior ability of polysaccharides to retain water, which may be attributed to the strong interactions between the hydrophilic groups in Alg and SF. All hydrogels exhibited considerable swelling capacity. The increase in ECM content in the structure increased the swelling rate. The highest water absorption rate was observed in the 5% ECM scaffold (81.06 ± 4.8). Studies showed that dECM‐derived scaffolds have high water absorption. This is because of the existence of hydrophilic groups. The results of our study were confirmed by other studies [55, 89].

The rate of scaffold destruction must be proportional to the bone tissue regeneration rate, which occurs in most bone tissue repair within 4 weeks [90]. The bone scaffold should not be completely destroyed before the repair phase is completed. The degradation rate of 3D‐printed constructs increased significantly with increasing the incubation time. The findings indicated that adding ECM to Alg‐SF hydrogel increased the degradability and improved the physiological stability. Increased degradation rate may be related to increased ECM content and hydrophobicity due to hydrogels' swelling ability. Scaffolds containing ECM lost approximately 24.53% ± 2.7 of their weight after 30 days of incubation, and this degradation rate seems suitable for bone tissue engineering [91].

Studies showed that ECM stiffness affects cell adhesion, proliferation, and differentiation [92]. Each tissue has its unique mechanical properties, and hydrogel matrices must mimic the stiffness of the desired tissue [93, 94]. One of the major challenges associated with ECM‐based bioinks is their poor mechanical performance compared to native bone tissue. We studied the effect of SF/Alg on composite hydrogels' tensile and compressive features. The average tensile strength indicated that SF/Alg content significantly impacted the tensile performance of hybrid hydrogels. This behavior was probably caused by the hydrogen bonding between Alg and SF, which produced a stiff hydrogel matrix [95]. Mohammadpour et al. [91] found that in alginate‐based nonsynthetic bio‐inks, increasing SF concentration could improve mechanical and biological properties and control the degradation rate [91]. The tensile and compressive strength of hydrogels increased markedly with increasing ECM content (*p* < 0.05) (3‐ and 2‐fold, respectively), which was in line with other studies [96]. This study found that hydrogels containing 5% ECM had the necessary mechanical properties for bone tissue engineering to create the physical matrix for bone regeneration.

The surface properties and noncytotoxicity of the scaffold are among the critical features that have an essential effect on the scaffold's ability to adherent and survive and differentiate cells [69, 97]. The cell viability rate on the hydrogel scaffolds was more than 80%, and the cell proliferation rate increased significantly with increasing ECM concentration. The effect of ECM in increasing adherence and cell survival was demonstrated in some studies [98, 99, 100]. Due to the presence of various proteins, ECM‐derived scaffolds allowed the cells to be more firmly attached to the matrix. So, more cells were attached. The results confirmed the 3D‐printed structures are nontoxic for mesenchymal cells and can create a controlled condition for the natural regeneration of bone tissue.

Based on the findings obtained from structural, physical and biological examinations, 5%ECM‐SF/Alg scaffold (as the optimized scaffold) was subjected to in vivo study. Studies of animal bone defect models help evaluate bone repair, and the most common and available animal models are usually mice, rabbits, and rats. The skull and femur have received more attention in examining bone defects in rats. In studies involving rat calvarial defects, an 8 mm‐sized defect is typically regarded as the critical size [101]. An 8 mm diameter (critical‐sized bone lesion) was successfully drilled on the left side of the skull. This defect cannot be repaired entirely during the lifetime of the animal. A bone defect of about 5–8 mm in rats is a CSD [102]. Joshi et al. [103] designed an Alg/SF‐based bio‐ink to differentiate human MSCs. They found that adding phosphate groups increased osteogenic differentiation while adding SF‐induced chondrogenic differentiation.

In general, very limited number of studies have been carried out on the impact of decellularized tissue‐derived structures on bone regeneration. Rameshbabu et al. [104] demonstrated that an amniotic sac‐derived scaffold can help cells to proliferate and osteogenic differentiation. The response of the host immune system to the implanted construct was observed along with the formation of blood vessels in vivo. In addition, the bone defect repaired 6 weeks after implantation was associated with high mineral density [104]. Dong et al. [105] studied an effective method to fabricate bone‐derived ECM‐polymer scaffolds to repair bone defects in a mouse skull model. Their findings showed that bone‐derived ECM/PCL scaffolds improved cell adhesion and facilitated bone expansion, proliferation and differentiation. Junka et al. fabricated electrospun osteoblast cells‐derived dECMs and PCL intending to enhance bone formation in cortical bone defects of mouse femurs. The results showed that their scaffold caused bone growth at the defect site and, together with vascular signs, stimulated endogenous cells for bone regeneration [106].

In the present study, histological examination in 4 weeks showed that bone tissue regeneration was observed in the printed scaffolds. However, connective tissue thickness was more significant in the 5% ECM‐SF/Alg group than in the other groups. No apparent new bone tissue was observed in the defects with no treatment. Compared with the samples implanted after 4 weeks, bone regeneration was improved 8 weeks postimplantation. In addition, Masson's trichrome and Alizarin red staining confirmed that the amount of collagen and calcium deposition in the 5%ECM‐SF/Alg group was considerably more significant than the SF/Alg group at week 4, which increased after 8 weeks. Following the staining results, a higher rate of ossification in the defects treated with 5%ECM‐SF/Alg for 4 and 8 weeks was confirmed by RT‐PCR assay.

ECM‐based scaffolds contain bioactive proteins, type IV collagen, proteoglycans, and functional proteins that bind to cytokines and growth factors that organize bone‐specific physiological and biochemical signals in connected cells [107]. In addition, cell–cell interactions promote the anchoring and homing of MSCs, osteoblasts, and osteoprogenitors [108]. These conditions lead to maintaining homeostasis and improving the survival capacity of these cells [49, 109]. These biochemical signals affect cell adhesion and fate [110]. In this way, these signals cause osteoblast cells to release TGFβ and VEGF, which leads to the migration of cells into the bone defect (differentiation of osteoblast cells) and vascular activation. Following the release of these factors, bone regeneration occurs with the formation of osteoblasts and the differentiation of osteocytes. Furthermore, regulation of immunity and polarization of macrophage response contribute to bone regeneration. Our findings showed that 5% of placenta tissue dECM blended in SF and Alg in printed form has significant potential as a biological scaffold in the organization of bone tissue, which can stimulate ossification and the formation of new bone tissue without growth factors.

## 5 Conclusion

Bone graft substitutes can be effectively manufactured by fabricating bioscaffolds using decellularization techniques. ECM‐based printed scaffolds contain all ECM compounds that perform a crucial function in simulating the microenvironment of native bone tissue and can regulate cell adhesion and tissue growth and repair. Incorporating ECM with natural polymers leads to increased mechanical properties to improve the clinical application of bone biografts. By increasing the concentration of ECM not only were the native topographic properties preserved but also the mechanical, biological, structural, and biochemical properties were improved. The ECM‐based scaffolds promoted cell–cell and matrix–cell interactions for new bone formation through biochemical signaling processes. Therefore, using dECM‐based printed structures is a promising strategy that performs a vital function in reconstructing significant bone defects. The immunogenic aspect of using dECM should be further considered to lead us to improve understanding of treatment approaches for bone lesions. Our investigations suggest SF/Alg 3D printed construct containing 5% w/w ECM as an optimized and excellent bone graft, and now promises to move toward clinical examinations.

## Author Contributions

M.G. and B.R. conceived, designed, and supervised the study. Z.B., Z. Khosrowpour, A.M., D.J., S.A., and H.N. performed the experiments. Z.B., H.P., Z. Khosrowpour, S.A., Z. Keshtkaran, M.A.‐A., H.P., S.S., and H.N. analyzed and interpreted the data. Z.B., Z. Khosrowpour, Z. Keshtkaran, D.J., S.A., H.P., F.B., and S.S. prepared the manuscript. M.G., B.R., and M.A.‐A. reviewed during the manuscript preparation and revised the manuscript.

## Ethics Statement

The examinations in the current study were conducted according to the guideline provided by the Ethical Committee of Larestan University of Medical Sciences, Larestan, Iran (Ethic code: IR.LARUMS.REC.1400.014).

## Conflicts of Interest

The authors declare no conflicts of interest.

## Data Availability

Data obtained from the current study are available from the corresponding author upon reasonable request.
